# Ethnic differences in preterm birth in Southwest China, 2014-2018: A population-based observational study

**DOI:** 10.3389/fmed.2022.972917

**Published:** 2022-08-04

**Authors:** Guiying Cao, Yanling Yuan, Cai Kong, Jue Liu, Min Liu, Hanfeng Ye

**Affiliations:** ^1^Department of Epidemiology and Biostatistics, School of Public Health, Peking University, Beijing, China; ^2^Yunnan Population and Family Planning Research Institute, Kunming, China

**Keywords:** preterm birth, delivery, ethnicity, difference, inequalities

## Abstract

**Objective:**

Preterm birth is a major healthcare problem and has been rising gradually in the past three decades in China. Yet the ethnic differences in the rates and distributions of preterm birth remain largely unknown in China. This study used data from Yunnan, a multiethnic province, to explore the differences in preterm birth across ethnicities.

**Methods:**

A population-based observational study was conducted based on data from the National Free Preconception Health Examination Project in rural Yunnan from Jan 1, 2014 to Dec 31, 2018. Pregnancies with at least one livebirth were included in this study. We estimated the rates and 95% confidence intervals (CIs) of overall preterm birth (born < 37 weeks’ gestation), moderate to late preterm birth (born between 32 and <37 weeks’ gestation), very preterm birth (born between 28 and 31 weeks’ gestation), and extremely preterm birth (born < 28 weeks’ gestation) across maternal ethnicity and compared them using log-binomial regressions. Multivariable log-binomial regressions were used to assess the association between maternal ethnicity and preterm birth with adjustment for potential confounders, including year of delivery, maternal age at delivery, education, occupation, pre-pregnancy body mass index, history of chronic disease, history of preterm birth, smoking and drinking alcohol during early pregnancy, and parity and multiple pregnancy of current pregnancy.

**Results:**

Among 195,325 women who delivered at least one live baby, 7.90% (95% CI, 7.78–8.02%) were born preterm. The rates of moderate to late preterm birth, very preterm birth, and extremely preterm birth were 6.20% (95% CI, 6.09–6.30%), 1.18% (95% CI, 1.13–1.23%), and 0.52% (95% CI, 0.49–0.56%), respectively. The rates of overall preterm birth, moderate to late preterm birth, very preterm birth, and extremely preterm birth differed across maternal ethnicity. The preterm birth rates in Dai (10.73%), Miao (13.23%), Lisu (12.64%), Zhuang (11.77%), Wa (10.52%), and Lagu (12.34%) women were significantly higher than that in Han women, and the adjusted relative risks were 1.45 [95% CI, 1.36, 1.54], 1.74 (95% CI, 1.62, 1.86), 1.60 (95% CI, 1.47, 1.75), 1.60 (95% CI, 1.46, 1.75), 1.40 (95% CI, 1.22, 1.60), and 1.67 (95% CI, 1.50, 1.87) respectively. There was no difference in preterm birth rate between Han women and Hani, Bai, or Hui women.

**Conclusion:**

This study found notable differences in the rates of preterm birth and its sub-categories across maternal ethnicities, which were especially higher in ethnic minority women. The findings suggest that greater efforts to reduce ethnic inequalities in preterm birth. Future studies are warranted to understand the drivers of ethnic inequalities in preterm birth in China.

## Introduction

Preterm birth is defined as babies born alive before 37 completed weeks of gestation or less than 259 days from the first date of a woman’s last menstrual period by the World Health Organization (WHO) (1, 2). Globally, it is estimated that 15 million babies are born preterm every year, and this number is rising (3, 4). The preterm birth rate was 10.6% in 2014 worldwide, ranging from 8.7 to 13.4% across regions (4). More than 80% of preterm births occur in Asia and sub-Saharan Africa, but preterm birth is truly a global problem (4). Complications of preterm birth are the leading cause of death in children younger than 5 years worldwide and were responsible for approximately 1 million deaths in 2015 (5). In addition, preterm neonates who survive are at greater risk of a range of short-term and long-term morbidities (6, 7). China has the second greatest number of preterm births after India worldwide, with more than 1 million babies born preterm every year (3, 4, 8). In China, the preterm birth rate increased by 1.1% per year from 1990 to 2016, with the highest increase in very preterm births born between 28 and 31 weeks gestation followed by late preterm births born between 34 and 36 weeks gestation, leading to a cause for concern (8, 9).

Preterm birth is considered to be a syndrome initiated by multiple mechanisms, including infection or inflammation, uteroplacental ischemia or hemorrhage, uterine overdistension, stress, and other immunologically mediated processes (10). The common factors associated with preterm birth include low and high maternal ages, multiple pregnancies, infections and chronic conditions such as diabetes and high blood pressure; however, a precise mechanism cannot be established in most cases (10). There could also be a genetic influence (10). In addition, several risk factors are known to be associated with preterm birth, including multiple medical and genetic causes and environmental and socioeconomic factors (11). One previous meta-analysis found that the risk of preterm birth varied by racial group: non-Hispanic black women had an increased risk of preterm birth than non-Hispanic white women (12). In addition, the risk of preterm birth also differed among the ethnic subgroups of Asian Americans in the United States, with increased odds in Filipinas and Vietnamese women but decreased odds in Chinese, Korean, Japanese, and Asian Indian women compared to non-Hispanic white women (13). In China, there are 55 ethnic minority groups, which represent one of the largest ethnic minority populations in the world (14). It was estimated that 114 million (8.5%) of the population in China belonged to ethnic minorities in 2000, and 71.4% of all ethnic minorities live in Western China (15, 16). Collectively, the 55 ethnic minority groups in China represent highly heterogeneous socioeconomic positions, languages, religions, and cultural and geographical contexts (16, 17). The majority of ethnic minorities in China live in remote, mountainous or nomadic areas with poor infrastructure and usually have economic and educational disadvantages (18, 19). The Chinese government has made substantial efforts to improve the rights and opportunities of ethnic minorities in China in the past 50 years (17). The government designated regions with large ethnic minority populations as “autonomous” and gave them the self-government right, including special legislative power and the right to develop and control local economies and finances. One previous meta-analysis reported that there were differences in maternal and child health outcomes and service coverage among ethnic minorities compared with Han populations in Western China (20). This study found that compared with Han women, ethnic minority women had lower odds of antenatal care use and birth in health facilities (20). For example, women of Hui ethnicity had lower odds of antenatal care and facility birth than Han women, and Tibetan and Uyghur ethnicities had lower odds of maternal and child health care coverage than the Han population (20). Thus, we hypothesize that there might be differences in preterm birth across ethnicities in China. However, no study has explored the preterm birth rate and associated factors across ethnicities in China.

The National Free Preconception Health Examination Project (NFPHEP) was launched by the Chinese National Health and Family Planning Commission and Ministry of Finance in 2010 to provide free health examinations and other services before conception for couples who planned to become pregnant in the next 6 months, as well as follow-up in their first trimester of pregnancy and after delivery in rural areas. In the current study, we used data from the NFPHEP in Yunnan, a multiethnic province in Southwest China, to estimate the differences in preterm birth rates in the Han ethnicity and individual ethnic groups.

## Materials and methods

### Study design and participants

We utilized data from the NFPHEP in rural Yunnan, China. In the NFPHEP, free preconception counseling, medical examinations, and management during pregnancy for all couples who had made their conception plan were provided by well-trained local health workers and obstetricians. Detailed information on the design, organization, and implementation of the NFPHEP has been described elsewhere (21, 22). Briefly, the baseline information of women who participated in the NFPHEP, including demographic characteristics (address of residence, ethnicity, birth date, age, education level, and occupation), history of chronic disease (anemia, hypertension, heart disease, diabetes, epilepsy, thyroid disease, chronic nephritis, cancer, tuberculosis, hepatitis B, and mental or psychological disorders), and history of pregnancy and adverse pregnancy outcomes (gravidity, parity, abortion, stillbirth, and preterm birth), were collected using a standardized questionnaire. Trained qualified local health workers then performed pre-pregnancy physical examinations for women to measure their bodyweight and height using calibrated instruments and take their blood samples. The information of women within 3 months after conception, including last menstrual period, cigarette consumption, and alcohol consumption during pregnancy, were collected by health workers through face-to-face interviews or by telephone. Information on current pregnancy outcomes (normal birth, preterm birth, miscarriage, induced abortion, or stillbirth), delivery date, gestational weeks, and newborn information (singleton or multiple births) was collected by health workers based on the medical records of the hospitals where delivery took place. Finally, the well-trained local health workers used data from the questionnaire, physical examination, and medical records to complete a standardized family heath file for all participants.

The study was terminated when women were enrolled by the NFPHEP in rural Yunnan and had pregnancy outcomes, such as normal birth, preterm birth, spontaneous abortion, induced abortion or stillbirth, between January 1, 2014, and December 31, 2018. This study was approved by the Institutional Review Board of the Chinese Association of Maternal and Child Health Studies and all participants provided written informed consent before enrollment.

### Outcomes

Preterm birth is the primary outcome of this study. We defined preterm birth as babies born alive before 37 weeks of pregnancy are completed according to the WHO (3, 23). Furthermore, the sub-categories of preterm birth based on gestational age included moderate to late preterm (32–<37 weeks), very preterm (28–31 weeks), and extremely preterm (less than 28 weeks) (3, 23). The proportions of liveborn preterm deliveries and its sub-categories were measured with respect to all deliveries in this study.

### Ethnic group

Analyses by ethnic groups were restricted to those with at least 1,000 participants and the ethnic groups with fewer than 1,000 participants were categorized into the “other” group. The ethnicity groups in this study were categorized into Han, Yi, Dai, Hani, Miao, Bai, Lisu, Zhuang, Hui, Wa, Lagu, and other.

### Other covariates

Maternal age at delivery was calculated based on the birth date and delivery date of women and was categorized into six groups: 18–24, 25–29, 30–34, 35–39, 40–44, and 45–49 years. Education level was categorized into primary school or below, junior high school, senior high school, and college and above. Occupation was categorized as farmer or worker. Pre-pregnancy body mass index (BMI) was calculated by dividing weight in kilograms by the square of height in meters and was categorized into four groups: <18.5 kg/m^2^ (underweight), 18.5–23.9 kg/m^2^ (normal weight), 24.0–27.9 kg/m^2^ (overweight), and ≥28.0 kg/m^2^ (obesity). Women were classified as having a history of chronic disease if they had one or more chronic diseases, including anemia, hypertension, heart disease, diabetes, epilepsy, thyroid disease, chronic nephritis, cancer, tuberculosis, hepatitis B, and mental or psychological disorders, whereas women were classified as not having a history of chronic disease if they did not have any of these diseases. Smoking status and drinking status during early pregnancy were both categorized as “yes” or “no” based on whether women smoked or drank alcohol during early pregnancy. Parity was divided into primiparous or multiparous based on current delivery. Type of pregnancy was grouped into singleton or multiple pregnancy based on current delivery.

### Statistical analysis

We summarized the maternal characteristics using the median and interquartile range (IQR) for continuous variables or frequencies (percentages) for categorized variables. The categorized characteristics of women across ethnicities were compared using Chi-square tests. We calculated the numbers and rates of preterm birth and sub-categories of preterm birth overall and separately according to maternal ethnicity. The rates with 95% confidence intervals (CIs) of preterm birth and each sub-category of preterm birth were calculated as the number of liveborn preterm deliveries divided by the total number of deliveries. Log-binomial regression models were used to estimate the crude risk ratios (cRRs) and 95% CIs of preterm births and sub-categories of preterm birth for maternal ethnicity. Multivariable log-binomial regression models were used to estimate the adjusted risk ratios (aRRs) with 95% CIs of preterm birth with maternal ethnicity with adjustment for potential confounders of preterm birth. All descriptive and inferential statistics were estimated using SAS 9.4 (SAS Institute, Inc., Cary, NC). Two-tailed p values less than 0.05 were considered statistically significant.

## Results

### Characteristics of participants

**FIGURE 1:**
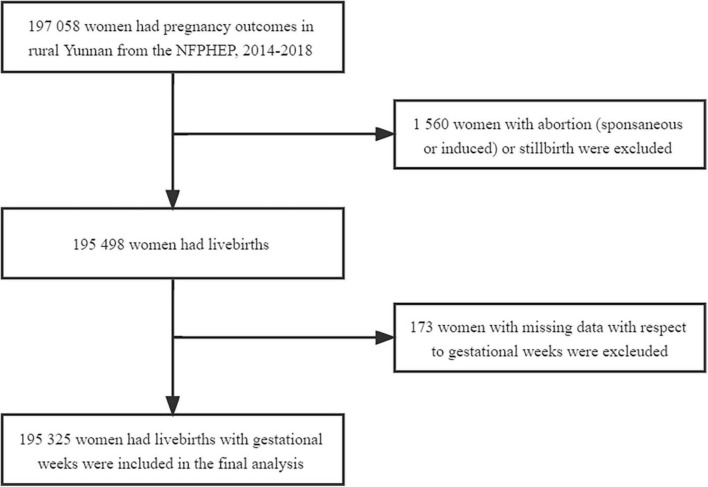
Study profile.

There were 197,058 women who had pregnancy outcomes at a reproductive age between 18 and 49 years in rural Yunnan between January 1, 2014, and December 31, 2018. We included 195,325 women aged 18–49 years women in rural Yunnan who delivered livebirths after excluding 1,560 women with abortion or stillbirth and 173 women with missing data with respect to gestational weeks in this period (Figure 1). The median age of all women included in the study was 26 years (IQR: 23–29). The characteristics of the women included in this study overall and by ethnicity are summarized in Table 1 and Supplementary Table 1. For the ethnicity of all women included in this study, approximately 62.62% (121,612) of women were of Han ethnicity, and Yi, Dai, Miao, Hani, Bai, Lisu, Zhuang, Lagu, and Wa ethnicity accounted for 15.12% (29,530), 4.50% (8,790), 2.87% (5,614), 2.84% (5,540), 2.26% (4,421), 1.90% (3,717), 1.84% (3,603), 1.07% (2,081), 0.91% (1,778), and 0.91% (1,775), respectively. The remaining 6,864 (3.51%) women were other ethnic minorities and had an ethnic group “other.” Among all women included in this study, 25.69% were aged 30 years and older at delivery. A total of 73.32% of them had an education level of junior high school or lower, and 52.62% of them were multiparous. Singleton pregnancies accounted for 99.46% of all pregnancies. There were significant differences in age at delivery, education level, occupation, pre-pregnancy BMI, history of chronic disease, status of smoking and drinking during early pregnancy, and parity and type of current pregnancy of women across ethnicities (*P* < 0.05). No difference in history of preterm birth was observed in women across ethnicities. The detailed frequencies and percentages of maternal sociodemographic characteristics, pre-pregnancy BMI, status of smoking and drinking during early pregnancy, history of chronic disease, history of preterm birth, and parity and type of current pregnancy by maternal ethnicity are summarized in Supplementary Table 1.

**TABLE 1:** Maternal characteristics with respect to ethnicities in rural Yunnan, Southwest China, 2014–2018.

Chara- cteristics	Total (*n* = 195,325)	Ethnicity												*P*-value

		Han (*n* = 121,612)	Yi (*n* = 29,530)	Dai (*n* = 8,790)	Hani (*n* = 5,614)	Miao (*n* = 5,540)	Bai (*n* = 4,421)	Lisu (*n* = 3,717)	Zhuang (*n* = 3,603)	Hui (*n* = 2,081)	Wa (*n* = 1,778)	Lagu (*n* = 1,775)	Other (*n* = 6,864)	
Total, %	100.00	62.26	15.12	4.50	2.87	2.84	2.26	1.90	1.84	1.07	0.91	0.91	3.51	
Year, %														<0.001
2014	13.86	13.72	16.86	13.32	13.06	11.91	9.93	16.01	15.82	10.09	9.22	6.87	10.88	
2015	14.90	14.66	17.12	14.23	14.41	16.08	8.78	16.79	11.63	12.35	17.72	11.04	15.47	
2016	18.89	18.95	19.61	19.34	17.46	16.66	17.12	21.87	15.76	16.00	20.75	18.31	18.71	
2017	23.95	24.15	22.16	24.29	22.21	25.27	28.21	20.26	27.01	29.22	24.47	28.62	22.79	
2018	28.40	28.52	24.24	28.82	32.86	30.07	35.96	25.07	29.78	32.34	27.84	35.15	32.15	
Maternal age at delivery (years),%														<0.001
18–24	32.84	31.60	33.62	31.47	31.87	45.90	24.75	42.96	41.10	44.50	34.76	39.15	33.22	
25–29	41.47	42.44	41.56	43.79	38.40	31.06	47.00	33.09	35.22	38.35	37.91	36.85	39.13	
30–34	17.88	18.05	17.33	18.57	20.34	14.96	20.11	15.60	16.65	11.82	19.01	16.34	19.09	
35–39	6.19	6.27	6.03	5.19	7.25	6.01	6.76	6.32	5.50	4.52	6.58	6.03	6.56	
41–44	1.51	1.54	1.35	0.92	1.98	1.86	1.29	1.88	1.47	0.67	1.57	1.63	1.82	
40–49	0.11	0.10	0.10	0.07	0.16	0.20	0.09	0.13	0.06	0.14	0.17	39.15	0.19	
Education, %														< 0.001
Primary school or below	18.35	11.89	23.26	30.01	30.10	51.66	7.26	51.06	17.90	9.23	54.95	49.97	34.95	
Junior high school	54.97	57.19	54.81	54.90	48.61	39.51	62.52	38.85	62.70	57.76	36.16	40.17	41.89	
Senior high school	14.21	16.46	12.52	9.47	13.77	5.29	8.78	5.14	11.57	18.16	5.46	5.07	8.45	
College and above	10.77	12.97	8.38	5.29	6.04	2.80	10.79	3.93	7.44	12.88	3.32	3.89	7.87	
Missing	1.70	1.50	1.03	0.33	1.48	0.74	10.65	1.02	0.39	1.97	0.11	0.90	6.85	
Occupation, %														<0.001
Farmer	91.78	90.36	94.94	97.11	94.19	96.39	89.10	94.86	92.01	89.81	96.68	96.85	88.83	
Worker	6.65	8.28	3.92	2.26	3.94	1.61	9.95	2.31	6.36	8.65	1.46	1.01	4.02	
Missing	1.57	1.36	1.14	0.63	1.87	2.00	0.95	2.82	1.64	1.54	1.86	2.14	7.15	
Pre-pregnancy BMI (kg/m^2^),%														<0.001
<18.5 (Underweight)	13.01	13.44	11.84	17.83	10.69	7.82	13.48	12.08	10.91	19.13	8.83	12.85	11.09	
18.5–23.9 (Normal weight)	70.20	70.14	71.17	64.08	68.77	75.34	67.04	69.36	77.91	66.41	74.02	67.94	71.23	
24.0–27.9 (Overweight)	13.54	13.44	13.29	14.06	15.66	14.28	14.27	15.63	9.33	11.48	14.45	14.65	14.19	
≥28.0 (Obesity)	3.24	2.99	3.70	4.03	4.88	2.56	5.20	2.93	1.86	2.98	2.70	4.56	3.50	
History of chronic disease, %	1.76	1.68	1.54	3.77	2.12	1.08	2.15	1.96	0.92	1.73	1.29	1.46	2.07	<0.001
History of preterm birth,%	0.36	0.35	0.39	0.50	0.30	0.31	0.32	0.40	0.17	0.24	0.62	0.39	0.32	0.171
Smokes during early pregnancy,%	1.06	1.25	0.70	0.77	0.98	0.49	0.90	0.67	0.31	0.72	1.24	0.79	0.82	<0.001
Drinks alcohol during early pregnancy,%	1.09	1.19	0.71	1.58	1.18	0.36	1.15	1.05	0.53	0.58	0.73	1.41	1.35	<0.001
Parity,%														<0.001
Primiparous	47.38	49.86	43.28	40.18	41.38	36.81	51.19	55.10	45.18	60.60	42.69	38.03	43.53	
Multiparous	52.62	50.14	56.72	59.82	58.62	63.19	48.81	55.10	54.82	39.40	57.31	61.97	56.47	
Type of pregnancy,%														0.037
Singleton	99.46	99.45	99.44	99.45	99.68	99.73	99.48	99.17	99.39	99.33	99.49	99.61	99.48	
Multiple	0.54	0.55	0.56	0.55	0.32	0.27	0.52	0.83	0.61	0.67	0.51	0.39	0.52	

### Rate of preterm birth by maternal ethnicity

The rates of preterm birth and sub-categories of preterm birth in overall and by ethnicity in 2014-2018 are presented in Table 2. The of preterm birth rate was 7.90% (95% CI, 7.78–8.02%) among the overall deliveries in rural Yunnan in 2014–2018. For the sub-categories of preterm birth, the rates were 6.20% (95% CI, 6.09–6.30%), 1.18% (95% CI, 1.13–1.23%), and 0.52% (95% CI, 0.49–0.56%) for moderate to late preterm birth, very preterm birth, and extremely preterm birth, respectively. The rate of preterm birth in Han women was 7.23% (95% CI, 7.08–7.37%), which was lower than that in Dai (10.73% [95% CI, 10.08–11.38%]), Miao (13.23% [95% CI, 12.34–14.12%]), Lisu (12.64% [95% CI, 11.58–13.71%]), Zhuang (11.77% [95% CI, 10.72–12.82%]), Wa (10.52% [95% CI, 9.09–11.94%]), Lagu (12.34% [95% CI, 10.81–13.87%]), and other minorities (11.01% [95% CI, 10.27–11.75%]) (*P* < 0.001) but higher than that in Yi (6.80% [95% CI, 6.51–7.08%]) (*P* = 0.001). There was no difference in preterm birth rates between Han women and Hani, Bai, or Hui women. For the rates of sub-categories of preterm birth across ethnicities, Yi women had a lower moderate to late preterm birth rate than Han women (*P* = 0.013); Bai women had a higher moderate to late preterm birth rate but a lower extremely preterm birth rate than Han women (*P* < 0.05); Dai, Zhuang, and Lagu women had a higher rate of moderate to late preterm birth and very preterm birth than Han women (*P* < 0.01); and Miao, Lisu, Wa, and other minority women had a higher rate of moderate to late preterm birth, very preterm birth, and extremely preterm birth than Han women (*P* < 0.001).

**TABLE 2:** Rates of preterm birth and sub-categories of preterm birth by maternal ethnicities in rural Yunnan, Southwest China, 2014–2018.

Ethnicity	Number of deliveries	Preterm birth				Moderate to late preterm birth				Very preterm birth				Extremely preterm birth			
					
		Number	Rate per 100 deliveries (95% CI)	cRR (95% CI)	*P*-value	Number	Rate per 100 deliveries (95% CI)	cRR (95% CI)	*P*-value	Number	Rate per 100 deliveries (95% CI)	cRR (95% CI)	*P*-value	Number	Rate per 100 deliveries (95% CI)	cRR (95% CI)	*P*-value
Total	195 325	15 429	7.90 (7.78, 8.02)			12 102	6.20 (6.09, 6.30)			2 303	1.18 (1.13, 1.23)			1 024	0.52 (0.49, 0.56)		
Han	121 612	8 791	7.23 (7.08, 7.37)	1 (ref)		6 939	5.71 (5.58, 5.84)	1 (ref)		1 268	1.04 (0.99, 1.10)	1 (ref)		584	0.48 (0.44, 0.52)	1 (ref)	
Yi	29 530	2 007	6.80 (6.51, 7.08)	0.94 (0.90, 0.98)	0.001	1 575	5.33 (5.08, 5.59)	0.93 (0.89, 0.99)	0.013	307	1.04 (0.92, 1.16)	1.00 (0.88, 1.13)	0.963	125	0.42 (0.35, 0.50)	0.88 (0.72, 1.06)	0.200
Dai	8 790	943	10.73 (10.08, 11.38)	1.48 (1.39, 1.58)	<0.001	774	8.81 (8.21, 9.40)	1.54 (1.44, 1.66)	<0.001	119	1.35 (1.11, 1.60)	1.30 (1.07, 1.56)	0.006	50	0.57 (0.41, 0.73)	1.18 (0.88, 1.56)	0.249
Hani	5 614	437	7.78 (7.08, 8.48)	1.08 (0.98, 1.18)	0.116	342	6.09 (5.47, 6.72)	1.07 (0.96, 1.18)	0.223	67	1.19 (0.91, 1.48)	1.14 (0.89, 1.45)	0.278	28	0.50 (0.31, 0.68)	1.04 (0.70, 1.49)	0.844
Miao	5 540	733	13.23 (12.34, 14.12)	1.83 (1.70, 1.96)	<0.001	533	9.62 (8.84, 10.04)	1.69 (1.55, 1.83)	<0.001	139	2.51 (2.10, 2.92)	2.41 (2.02, 2.85)	<0.001	61	1.10 (0.83, 1.38)	2.29 (1.75, 2.95)	<0.001
Bai	4 421	334	7.55 (6.78, 8.37)	1.05 (0.94, 1.16)	0.410	284	6.42 (5.70, 7.15)	1.13 (1.00, 1.26)	0.043	40	0.90 (0.63, 1.18)	0.87 (0.62, 1.17)	0.375	10	0.23 (0.09, 0.37)	0.47 (0.24, 0.83)	0.018
Lisu	3 717	470	12.64 (11.58, 13.71)	1.75 (1.60, 1.91)	<0.001	338	9.09 (8.17, 10.02)	1.59 (1.43, 1.77)	<0.001	87	2.34 (1.85, 2.83)	2.24 (1.80, 2.76)	<0.001	45	1.21 (0.86, 1.56)	2.52 (1.84, 3.37)	<0.001
Zhuang	3 603	424	11.77 (10.72, 12.82)	1.63 (1.48, 1.78)	<0.001	324	8.99 (8.06, 9.93)	1.58 (1.41, 1.75)	<0.001	79	2.19 (1.71, 2.67)	2.10 (1.67, 2.61)	<0.001	21	0.58 (0.33, 0.83)	1.21 (0.76, 1.82)	0.382
Hui	2 081	128	6.15 (5.13, 7.18)	0.85 (0.71, 1.00)	0.061	102	4.90 (3.97, 5.83)	0.86 (0.71, 1.03)	0.118	21	1.01 (0.58, 1.44)	0.97 (0.61, 1.45)	0.881	5	0.24 (0.03, 0.45)	0.50 (0.18, 1.08)	0.123
Wa	1 778	187	10.52 (9.09, 11.94)	1.45 (1.26, 1.66)	<0.001	131	7.37 (6.15, 8.58)	1.29 (1.09, 1.52)	0.003	36	2.02 (1.37, 2.68)	1.94 (1.37, 2.65)	<0.001	20	1.12 (0.63, 1.62)	2.34 (1.45, 3.54)	<0.001
Lagu	1 775	219	12.34 (10.81, 13.87)	1.71 (1.50, 1.93)	<0.001	165	9.30 (7.94, 10.65)	1.63 (1.40, 1.88)	<0.001	40	2.25 (1.56, 2.94)	2.16 (1.56, 2.91)	<0.001	14	0.79 (0.38, 1.20)	1.64 (0.92, 2.67)	0.066
Other	6 864	756	11.01 (10.27, 11.75)	1.52 (1.42, 1.63)	<0.001	595	8.67 (8.00, 9.33)	1.52 (1.40, 1.64)	<0.001	100	1.46 (1.17, 1.74)	1.40 (1.13, 1.70)	0.001	61	0.89 (0.67, 1.11)	1.85 (1.41, 2.38)	<0.001

cRR, crude risk ratio.

**FIGURE 2:**
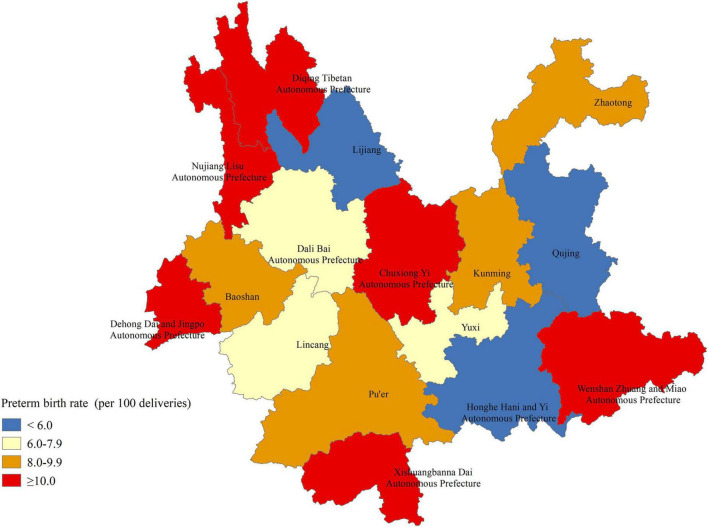
Geographic disparities in the rates of preterm birth in rural Yunnan, Southwest China, 2014–2018.

According to the concentration of ethnic minorities in Yunnan, the rates of preterm birth were also marked in maps to clearly show the ethnic and regional disparities in preterm birth (Figure 2). At the municipal/ethnic autonomous prefecture level, the preterm birth rate was higher than 10.0% in six autonomous prefectures for ethnic minorities, including the Diqing Tibetan Autonomous Prefecture (17.00%), Wenshan Zhuang and Miao Autonomous Prefecture (14.68%), Xishuangbanna Dai Autonomous Prefecture (14.65%), Nujiang Lisu Autonomous Prefecture (12.50%), Chuxiong Yi Autonomous Prefecture (12.08%), and Dehong Dai and Jingpo Autonomous Prefecture (10.66%). In Diqing Tibetan Autonomous Prefecture, the Lisu minority accounted for 34.32% after the “other” minority group (39.45%) (Supplementary Table 2). In Xishuangbanna, approximately 54% of women with livebirths were of the Dai, Miao, Lisu, Zhuang, Lagu, or Wa ethnicity (Supplementary Table 2). The rates of preterm birth were 4.62% and 7.13% in Honghe Hani and Yi Autonomous Prefecture and Dali Bai Autonomous Prefecture, respectively. The rates of preterm birth were low in other municipal/ethnic autonomous prefectures dominated by Han and Yi ethnicities, such as Qujing (3.91%), Yuxi (7.29%), and Lijiang (5.81%) (Figure 2 and Supplementary Table 2).

**FIGURE 3:**
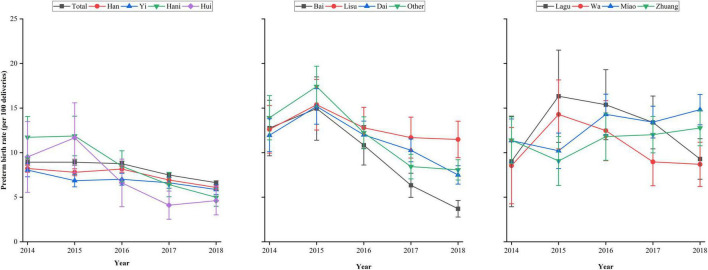
Preterm birth rates across ethnicities in rural Yunnan, Southwest China, from 2014 to 2018.

Figure 3 and Supplementary Table 3 show the preterm birth rates across maternal ethnicities from 2014 to 2018. Overall, the preterm birth rate decreased from 8.93% (95% CI, 8.59–9.27%) in 2014 to 6.63% (95% CI, 6.42–6.83%) in 2018. A decreased rate of preterm birth from 2014 to 2018 was observed in Han, Yi, Hani, Hui, Bai, Lisu, Dai, and other ethnic minorities categorized as “other” women. However, the preterm birth rate slightly increased from 9.02% (95% CI, 3.93–14.10%) in 2014 to 9.29% (95% CI, 7.02–11.57%) in 2018 in Lagu women and from 8.54% (95% CI, 4.26–12.81%) in 2014 to 8.69% (95% CI, 6.21–11.17%) in 2018 in Wa women. Among Miao and Zhuang women, the rates of preterm birth increased from 11.36% (95% CI, 8.94–13.78%) in 2014 to 14.83% (95% CI, 13.12–16.53%) in 2018 and 11.40% (95% CI, 8.79–14.01%) in 2014 to 12.77% (95% CI, 10.77–14.76%) in 2018, respectively.

**FIGURE 4:**
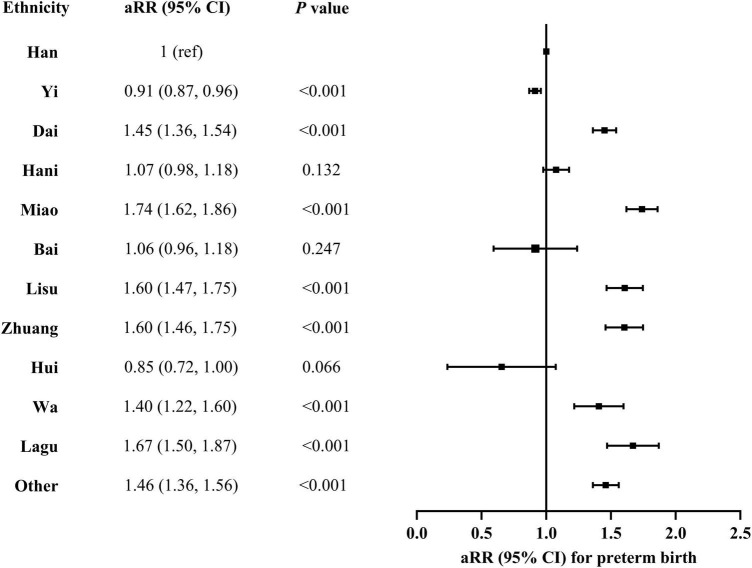
Association between preterm birth and maternal ethnicity in rural Yunnan, Southwest China, 2014–2018. aRR, adjusted risk ratio. A multivariable log-binominal regression model was conducted with adjustment for year of delivery, maternal age at delivery, education, occupation, pre-pregnancy, history of chronic disease, history of preterm birth, smoking and drinking alcohol during early pregnancy, and parity and multiple pregnancy of current pregnancy.

In the multivariable regression models adjusted for maternal sociodemographic characteristics, pre-pregnancy BMI, status of smoking and drinking alcohol during early pregnancy, history of chronic disease, history of preterm birth, and parity and type of current pregnancy, preterm birth was significantly associated with ethnicity among women with livebirth in rural Yunnan (Figure 4). Compared with Han ethnicity, Yi ethnicity had a decreased risk of preterm birth (aRR = 0.91; 95% CI, 0.87, 0.96). There was an increased risk of preterm birth in Dai (aRR = 1.45; 95% CI, 1.36, 1.54), Miao (aRR = 1.74; 95% CI, 1.62, 1.86), Lisu (aRR = 1.60; 95% CI, 1.47, 1.75), Zhuang (aRR = 1.60; 95% CI, 1.46, 1.75), Wa (aRR = 1.40; 95% CI, 1.22, 1.60), and other minorities categorized as “other” (aRR = 1.46; 95% CI, 1.36, 1.56). There was no difference in preterm birth between Han ethnicity and Hani, Bai, or Hui ethnicity.

## Discussion

Preterm birth is a major healthcare problem affecting 15 million births worldwide every year. We performed a population-based, observational study in rural Yunnan, China, to estimate the rates of preterm birth and sub-categories preterm birth as well as the ethnicity differences in preterm birth. We found that the preterm birth rate was 7.90% in rural Yunnan in 2014–2018, and the rates of sub-categories preterm birth were 6.20% for moderate to late preterm birth, 1.18% for very preterm birth, and 0.52% for extremely preterm birth in this period. The overall preterm birth rate decreased from 8.93% in 2014 to 6.63% in 2018; however, it increased in Lagu (from 9.02 to 9.29%), Wa (from 8.54 to 8.69%), Miao (from 11.36 to 14.83%), and Zhuang (from 11.40 to 12.77%) women from 2014 to 2018. This study found significant differences in rates of preterm birth and sub-categories preterm birth across maternal ethnicity. After adjusting for potential confounders, Yi women had a decreased risk of preterm birth compared with Han women, and Dai, Miao, Lisu, Zhuang, and Wa women had an increased risk of preterm birth compared with Han women.

The preterm birth rate in rural Yunnan reported in our study (7.90%) was slightly higher than the pooled rate (7.04%) estimated for China in 2015–2016 reported by one previous meta-analysis (9). In addition, this meta-analysis found that the preterm birth rate varied from 3.8 to 7.3% across administration regions in China in 1990–2016, with the highest rate in the Northwest (7.3%) followed by the Southwest (6.96%) and the East (6.19%) (9). Similar to our preterm birth rate in rural Yunnan (7.90%), one previous study based on 42 139 neonates born in the department of obstetrics in 77 hospitals in 16 provinces in China reported that the preterm birth rate was 7.8% in 2002–2003 (24), and another one previous study conducted a multicentre cross-sectional survey involving 89 hospitals in 25 provinces in China and reported preterm birth rate of 7.3% in 2015–2016 (25). However, Zhu and colleagues (26) reported a substantially higher rate of preterm birth (11.0%) of singleton livebirths from 63 tertiary hospitals in 23 provinces in China between 2011 and 2014 than the rate in rural Yunnan (7.90%). Additionally, using data from the NFPHEP, Liu and colleagues (22) found a lower rate of preterm birth (5.26%) among singleton livebirths in rural areas of 31 provinces in China in 2011–2012 than the rate in rural Yunnan reported in our study. In addition, the preterm birth rate (7.90%) in rural Yunnan reported in the current study was higher than the rate (6.1%) estimated using national monitoring data of more than 9 million women from China’s National Maternal Near Miss Surveillance System in 2012–2018 (27). Zou and colleagues (28) conducted a multicentre survey enrolling 107 905 livebirths from 14 provinces in China and found that the preterm birth rate was 7.1% in 2011, which was slightly lower than the rate reported in our study. Several possible reasons for the varied preterm birth rates between studies included differences in administration region (province level data vs. national level data), data source (population-based data vs. hospital-based data), definition of preterm birth (<37 weeks vs. 28 to <37 weeks), and calculation of preterm birth (among all livebirths vs. singleton livebirths). Preterm birth is officially defined as a delivery between 28 and <37 completed weeks of gestation in China (29). Several previous studies reported that the rate of extremely preterm birth (less than 28 weeks) ranged from 0.2 to 0.4% in high-income countries (30–33). In this study, we found that the rate of extremely preterm birth (<28 weeks gestation) was 0.52% in rural Yunnan in 2014–2018. In recent decades, China has achieved great advances in neonatal care, and the survival rate of extremely preterm births has reached a respectable level, exceeding 80% in neonates born at 26 weeks gestation (8, 34). It is time for China to consider redefining preterm birth as babies born from 24 weeks to <36 weeks of gestation, to make the definition of preterm birth more comparable with high-income countries (8).

There are few studies exploring the distribution of preterm birth and sub-categories of preterm birth in ethnic minorities and Han women in China. This study using data from NFPHEP in Yunnan, a multiethnic province in Western China, found significant differences in rates of preterm birth and sub-categories preterm birth across maternal ethnicity. We found that the preterm birth rates ranged from 6.15 to 13.23% across maternal ethnicities, with the highest rate in Miao women (13.23%) followed by Lisu (12.64%) and Lagu (12.34%). We also found that the trends in preterm birth rates from 2014 to 2018 differed across maternal ethnicity and an increased rate was observed in Lagu, Wa, Miao, and Zhuang women. In addition, there were also differences in the rates of sub-categories of preterm birth across maternal ethnicities, with the highest rates of both moderate to late preterm birth (9.62%) and very preterm birth (2.51%) observed in Miao women and the highest rate of extremely preterm birth observed in Lagu women (1.21%). The preterm birth rates of several ethnic minorities in rural Yunnan, such as Miao (13.23%), Lisu (12.64%), and Lagu (12.34%), were much higher than the global rate (10.6%) in 2014 (4). After adjusting for potential confounders of preterm birth, significant differences were also observed across maternal ethnicity, with a higher risk of preterm birth in Dai, Miao, Lisu, Zhuang, Wa, Lagu, and other ethnic minorities grouped as ‘other’ women than Han women, but a lower risk of preterm birth in Yi women than Han women. There are 56 ethnic groups in China, with Chinese Han being the majority. There are substantial differences in genetic background, culture, socioeconomic levels, climate and geographic features of the residential area, lifestyle, and dietary pattern among certain ethnic groups (35). In China, ethnic minorities have some privileges such as looser family planning restrictions and educational benefits. Fertility controls have been less stringent for people living in autonomous regions and many ethnic minority couples were allowed a second or third child under the one-child policy before the second- and three-child policy in China (36). Although the Chinese government has made substantial efforts to improve the rights and opportunities of its ethnic minorities in the past 50 years, there are still differences in maternal and child health care coverage between ethnic minorities and Han populations in in west China (20). Compared with Han women, ethnic minority women had lower odds of antenatal care use and birth in health facilities in west China (20). Much of the literature on the ethnic differences in preterm birth draws from women in the United States, including non-Hispanic black women, non-Hispanic white women, Hispanic women, and Asian American women (12, 13, 37). The weathering hypothesis has been proposed as a possible explanation for the differences in preterm birth among African-American women and non-Hispanic white women, which posits chronic exposure to psychosocial and environmental stressors prematurely ages exposed African-American women, shifting age-related risk to the peak of reproductive activity (38, 39). However, it remains uncertain whether the weathering hypothesis can be proposed as a possible explanation for the ethnic differences in preterm birth in China. Future studies are warranted to explore the associated factors of the differences in preterm birth across ethnicities in China.

The large sample size of this study provided, to our knowledge, the first direct comparison of preterm birth rates among major minority groups in Western China within one survey, using the definition of preterm birth and its sub-categories according to the WHO. However, some limitations in this study should also be acknowledged. First, this study was conducted in Yunnan, a multiethnic province in Southwest China, which differs from other provinces in population structure and ethnic composition, perhaps leading to limited national representativeness. Thus, future studies are warranted to further explore the association between ethnicity and preterm birth in China to achieve the Millennium Development Goal for maternal and reproductive health. Second, this study was unable to separate spontaneous preterm births from iatrogenic preterm births because the mode of delivery was simply grouped into vaginal or cesarean delivery in the NFPHEP. Third, several factors possibly associated with preterm birth were not included in this study, such as environmental and nutritional factors, socioeconomic status, and lifestyle.

In summary, we found that the preterm birth rates ranged from 6.15 to 13.23% across maternal ethnicities in rural Yunnan. Notable differences in rates of preterm birth and its sub-categories were observed across maternal ethnicity, especially higher in ethnic minority women. We recommend further research to understand the drivers of ethnic inequalities in preterm birth, together with policy changes to improve the equity of access to maternal and child health services for women from minority ethnic groups in China.

## Data availability statement

The original contributions presented in this study are included in the article/Supplementary material, further inquiries can be directed to the corresponding authors.

## Ethics statement

This study was approved by the Institutional Review Board of the Chinese Association of Maternal and Child Health Studies and all participants provided written informed consent before enrollment.

## Author contributions

GC and ML searched the literature, designed the study, analyzed the data, interpreted the results, and drafted the manuscript. YY, CK, and HY collected the data, interpreted the results, and revised the manuscript. GC, JL, and ML conceived the study, designed the study, supervised the study, interpreted the results, and revised the manuscript. All authors approved the final version of the manuscript.
