# Supplementary material for: Ethnic differences in preterm birth in Southwest China, 2014-2018: A population-based observational study
Source: Front Med (Lausanne). 2022 Aug 4;9:972917. doi: 10.3389/fmed.2022.972917 (PMC9386050; doi:10.3389/fmed.2022.972917)
Supplement: Supplementary file 1 [file Table_1.pdf]

eTable 1. Maternal characteristics with respect to ethnicities in rural Yunnan, Southwest China, 2014-2018

Characteristics	Total	Ethnicity											
		Han	Yi	Dai	Hani	Miao	Bai	Lisu	Zhuang	Hui	Wa	Lagu	Other
Total, n(%)	195 325(100)	121 612(62.26)	29 530(15.12)	8 790(4.50)	5 614(2.87)	5 540(2.84)	4 421(2.26)	3 717(1.90)	3 603(1.84)	2 081(1.07)	1 778(0.91)	1 775(0.91)	6 864(3.51)
Year													
2014	27 078(13.86)	16 687(13.72)	4 980(16.86)	1 171(13.32)	733(13.06)	660(11.91)	439(9.93)	595(16.01)	570(15.82)	210(10.09)	164(9.22)	122(6.87)	747(10.88)
2015	29 096(14.90)	17 829(14.66)	5 055(17.12)	1 251(14.23)	809(14.41)	891(16.08)	388(8.78)	624(16.79)	419(11.63)	257(12.35)	315(17.72)	196(11.04)	1 062(15.47)
2016	36 891(18.89)	23 047(18.95)	5 792(19.61)	1 700(19.34)	980(17.46)	923(16.66)	757(17.12)	813(21.87)	568(15.76)	333(16.00)	369(20.75)	325(18.31)	1 284(18.71)
2017	46 780(23.95)	29 365(24.15)	6 545(22.16)	2 135(24.29)	1 247(22.21)	1 400(25.27)	1 247(28.21)	753(20.26)	973(27.01)	608(29.22)	435(24.47)	508(28.62)	1 564(22.79)
2018	55 480(28.40)	34 684(28.52)	7 158(24.24)	2 533(28.82)	1 845(32.86)	1 666(30.07)	1 590(35.96)	932(25.07)	1 073(29.78)	673(32.34)	495(27.84)	624(35.15)	2 207(32.15)
Maternal age at delivery (years), n(%)													
18-24	64 144(32.84)	38 427(31.60)	9 928(33.62)	2 766(31.47)	1 789(31.87)	2543(45.90)	1 094(24.75)	1 597(42.96)	1 481(41.10)	926(44.50)	618(34.76)	695(39.15)	2 280(33.22)
25-29	80 999(41.47)	51 612(42.44)	12 272(41.56)	3 849(43.79)	2 156(38.4)	1 721(31.06)	2 078(47)	1 230(33.09)	1 269(35.22)	798(38.35)	674(37.91)	654(36.85)	2 686(39.13)
30-34	34 932(17.88)	21 957(18.05)	5 119(17.33)	1 632(18.57)	1 142(20.34)	829(14.96)	889(20.11)	580(15.6)	600(16.65)	246(11.82)	338(19.01)	290(16.34)	1 310(19.09)
35-39	12 099(6.19)	7 621(6.27)	1 782(6.03)	456(5.19)	407(7.25)	333(6.01)	299(6.76)	235(6.32)	198(5.5)	94(4.52)	117(6.58)	107(6.03)	450(6.56)
40-44	2 942(1.51)	1 871(1.54)	400(1.35)	81(0.92)	111(1.98)	103(1.86)	57(1.29)	70(1.88)	53(1.47)	14(0.67)	28(1.57)	29(1.63)	125(1.82)
45-49	209(0.11)	124(0.10)	29(0.10)	6(0.07)	9(0.16)	11(0.20)	4(0.09)	5(0.13)	2(0.06)	3(0.14)	3(0.17)	695(39.15)	13(0.19)
Education, n(%)													
Primary school or below	35 837(18.35)	14 460(11.89)	6 868(23.26)	2 638(30.01)	1 690(30.10)	2 862(51.66)	321(7.26)	1 898(51.06)	645(17.90)	192(9.23)	977(54.95)	887(49.97)	2 399(34.95)
Junior high school	107 379(54.97)	69 549(57.19)	16 186(54.81)	4 826(54.90)	2 729(48.61)	2 189(39.51)	2 764(62.52)	1 444(38.85)	2 259(62.70)	1 202(57.76)	643(36.16)	713(40.17)	2 875(41.89)
Senior high school	27 749(14.21)	20 012(16.46)	3 698(12.52)	832(9.47)	773(13.77)	293(5.29)	388(8.78)	191(5.14)	417(11.57)	378(18.16)	97(5.46)	90(5.07)	580(8.45)
College and above	21 030(10.77)	15 770(12.97)	2 474(8.38)	465(5.29)	339(6.04)	155(2.80)	477(10.79)	146(3.93)	268(7.44)	268(12.88)	59(3.32)	69(3.89)	540(7.87)
Missing	3 330(1.70)	1 821(1.50)	304(1.03)	29(0.33)	83(1.48)	41(0.74)	471(10.65)	38(1.02)	14(0.39)	41(1.97)	2(0.11)	16(0.9)	470(6.85)
Occupation, n(%)													
Farmer	179 274(91.78)	109 891(90.36)	28 035(94.94)	8 536(97.11)	5 288(94.19)	5 340(96.39)	3 939(89.10)	3 526(94.86)	3 315(92.01)	1 869(89.81)	1 719(96.68)	1 719(96.85)	6 097(88.83)
Worker	12 989(6.65)	10 068(8.28)	1 157(3.92)	199(2.26)	221(3.94)	89(1.61)	440(9.95)	86(2.31)	229(6.36)	180(8.65)	26(1.46)	18(1.01)	276(4.02)
Missing	3 062(1.57)	1 653(1.36)	338(1.14)	55(0.63)	105(1.87)	111(2.00)	42(0.95)	105(2.82)	59(1.64)	32(1.54)	33(1.86)	38(2.14)	491(7.15)
Pre-pregnancy BMI (kg/m^2), n(%)													
<18.5 (Underweight)	25 419(13.01)	16 341(13.44)	3 496(11.84)	1 567(17.83)	600(10.69)	433(7.82)	596(13.48)	449(12.08)	393(10.91)	398(19.13)	157(8.83)	228(12.85)	761(11.09)
18.5-23.9 (Normal weight)	13 7124(70.20)	85 297(70.14)	21 017(71.17)	5 633(64.08)	3 861(68.77)	4 174(75.34)	2 964(67.04)	2 578(69.36)	2 807(77.91)	1 382(66.41)	1 316(74.02)	1 206(67.94)	4 889(71.23)
24.0-27.9 (Overweight)	26 452(13.54)	16 343(13.44)	3 925(13.29)	1 236(14.06)	879(15.66)	791(14.28)	631(14.27)	581(15.63)	336(9.33)	239(11.48)	257(14.45)	260(14.65)	974(14.19)

≥28.0 (Obesity)	6 330(3.24)	3 631(2.99)	1 092(3.7)	354(4.03)	274(4.88)	142(2.56)	230(5.20)	109(2.93)	67(1.86)	62(2.98)	48(2.70)	81(4.56)	240(3.50)
History of chronic disease, n(%)													
No	191 887(98.24)	119 566(98.32)	29 076(98.46)	8 459(96.23)	5 495(97.88)	5 480(98.92)	4 326(97.85)	3 644(98.04)	3 570(99.08)	2 045(98.27)	1755(98.71)	1 749(98.54)	6 722(97.93)
Yes	3 438(1.76)	2046(1.68)	454(1.54)	331(3.77)	119(2.12)	60(1.08)	95(2.15)	73(1.96)	33(0.92)	36(1.73)	23(1.29)	26(1.46)	142(2.07)
History of preterm birth, n(%)													
No	194 624(99.64)	121 183(99.65)	29 416(99.61)	8 746(99.50)	5 597(99.70)	5523(99.69)	4 407(99.68)	3 702(99.60)	3 597(99.83)	2 076(99.76)	1 749(98.54)	1 768(99.61)	6 842(99.68)
Yes	701(0.36)	429(0.35)	114(0.39)	44(0.50)	17(0.30)	17(0.31)	14(0.32)	15(0.40)	6(0.17)	5(0.24)	26(1.46)	7(0.39)	22(0.32)
Smokes during early pregnancy, n(%)													
No	193 264(98.94)	120 090(98.75)	29324(99.30)	8 722(99.23)	5 559(99.02)	5 513(99.51)	4 381(99.1)	3 692(99.33)	3 592(99.69)	2 066(99.28)	1 756(98.76)	1 761(99.21)	6 808(99.18)
Yes	2061(1.06)	1522(1.25)	206(0.70)	68(0.77)	55(0.98)	27(0.49)	40(0.9)	25(0.67)	11(0.31)	15(0.72)	22(1.24)	14(0.79)	56(0.82)
Drinks alcohol during early pregnancy, n(%)													
No	193 190(98.91)	120 165(98.81)	29 319(99.29)	8 651(98.42)	5 548(98.82)	5 520(99.64)	4 370(98.85)	3 678(98.95)	3 584(99.47)	2 069(99.42)	1 765(99.27)	1 750(98.59)	6 771(98.65)
Yes	2135(1.09)	1447(1.19)	211(0.71)	139(1.58)	66(1.18)	20(0.36)	51(1.15)	39(1.05)	19(0.53)	12(0.58)	13(0.73)	25(1.41)	93(1.35)
Parity, n(%)													
Primiparous	92 552(47.38)	60 635(49.86)	12 780(43.28)	3 532(40.18)	2 323(41.38)	2 039(36.81)	2 263(51.19)	1 669(44.90)	1 628(45.18)	1 261(60.60)	759(42.69)	675(38.03)	2 988(43.53)
Multiparous	102 773(52.62)	60 977(50.14)	16 750(56.72)	5 258(59.82)	3 291(58.62)	3 501(63.19)	2 158(48.81)	2 048(55.10)	1 975(54.82)	820(39.40)	1 019(57.31)	1 100(61.97)	3 876(56.47)
Type of pregnancy, n(%)													
Singleton	194 272(99.46)	12 0947(99.45)	29 365(99.44)	8 742(99.45)	5 596(99.68)	5 525(99.73)	4 398(99.48)	3 686(99.17)	3 581(99.39)	2 067(99.33)	1 769(99.49)	1 768(99.61)	6 828(99.48)
Multiple	1 053(0.54)	665(0.55)	165(0.56)	48(0.55)	18(0.32)	15(0.27)	23(0.52)	31(0.83)	22(0.61)	14(0.67)	9(0.51)	7(0.39)	36(0.52)

eTable 2. The maternal ethnicity by municipal/ethnic autonomous prefecture level in rural Yunnan, Southwest China, 2014-2018

Municipal/ethnic autonomous prefecture	Total, n(%)	Ethnicity											
		Han, n(%)	Yi, n(%)	Dai, n(%)	Hani, n(%)	Miao, n(%)	Bai, n(%)	Lisu, n(%)	Zhuang, n(%)	Hui, n(%)	Wa, n(%)	Lagu, n(%)	Other, n(%)
Kunming	13 627(100.00)	11 432(83.89)	1 299(9.53)	39(0.29)	62(0.45)	163(1.20)	176(1.29)	50(0.37)	40(0.29)	278(2.04)	7(0.05)	8(0.06)	73(0.54)
Qujing	25 015(100.00)	23 624(94.44)	506(2.02)	17(0.07)	32(0.13)	131(0.52)	21(0.08)	9(0.04)	137(0.55)	182(0.73)	10(0.04)	5(0.02)	341(1.36)
Yuxi	18 808(100.00)	12 794(68.02)	3 974(21.13)	623(3.31)	680(3.62)	74(0.39)	80(0.43)	6(0.03)	26(0.14)	296(1.57)	3(0.02)	85(0.45)	167(0.89)
Baoshan	8 074(100.00)	7 054(87.37)	278(3.44)	177(2.19)	4(0.05)	40(0.50)	76(0.94)	124(1.54)	2(0.02)	21(0.26)	26(0.32)	2(0.02)	270(3.34)
Zhaotong	10 578(100.00)	9 577(90.54)	175(1.65)	3(0.03)	6(0.06)	286(2.70)	4(0.04)	3(0.03)	11(0.1)	378(3.57)	2(0.02)	3(0.03)	130(1.23)
Lijiang	8 978(100.00)	3 620(40.32)	3 333(37.12)	81(0.9)	4(0.04)	44(0.49)	127(1.41)	837(9.32)	29(0.32)	16(0.18)	5(0.06)	1(0.01)	881(9.81)
Pu'er	9 568(100.00)	3 196(33.40)	2 028(21.2)	388(4.06)	2 184(22.83)	50(0.52)	34(0.36)	11(0.11)	12(0.13)	50(0.52)	331(3.46)	1 053(11.01)	231(2.41)
Lincang	10 296(100.00)	5 780(56.14)	1 849(17.96)	563(5.47)	17(0.17)	51(0.50)	105(1.02)	57(0.55)	10(0.1)	34(0.33)	1 312(12.74)	235(2.28)	283(2.75)
Chuxiong Yi Autonomous Prefecture	11 197(100.00)	7 600(67.88)	2972(26.54)	58(0.52)	46(0.41)	226(2.02)	78(0.7)	92(0.82)	12(0.11)	32(0.29)	5(0.04)	5(0.04)	71(0.63)
Honghe Hani and Yi Autonomous Prefecture	32 732(100.00)	16 987(51.9)	9 758(29.81)	979(2.99)	1639(5.01)	1923(5.87)	38(0.12)	13(0.04)	405(1.24)	465(1.42)	15(0.05)	88(0.27)	422(1.29)
Wenshan Zhuang and Miao Autonomous Prefecture	13 556(100.00)	6 499(47.94)	1 101(8.12)	120(0.89)	27(0.2)	2 412(17.79)	60(0.44)	3(0.02)	2 903(21.41)	33(0.24)	6(0.04)	3(0.02)	389(2.87)
Xishuangbanna Dai Autonomous Prefecture	4 759(100.00)	535(11.24)	315(6.62)	2 211(46.46)	889(18.68)	44(0.92)	11(0.23)	1(0.02)	2(0.04)	16(0.34)	35(0.74)	271(5.69)	429(9.01)
Dali Bai Autonomous Prefecture	14 956(100.00)	9407(62.9)	1 784(11.93)	32(0.21)	20(0.13)	52(0.35)	2 999(20.05)	110(0.74)	8(0.05)	261(1.75)	5(0.03)	16(0.11)	262(1.75)
Dehong Dai and jingpo Autonomous Prefecture	9 028(100.00)	3 093(34.26)	23(0.25)	3 491(38.67)	2(0.02)	6(0.07)	30(0.33)	307(3.4)	4(0.04)	11(0.12)	14(0.16)	0(0)	2 047(22.67)
Nujiang Lisu Autonomous Prefecture	2 224(100.00)	147(6.61)	35(1.57)	6(0.27)	1(0.04)	3(0.13)	490(22.03)	1 432(64.39)	2(0.09)	1(0.04)	0(0)	0(0)	107(4.81)
Diqing Tibetan Autonomous Prefecture	1 929(100.00)	267(13.84)	100(5.18)	2(0.10)	1(0.05)	35(1.81)	92(4.77)	662(34.32)	0(0)	7(0.36)	2(0.1)	0(0)	761(39.45)

eTable 3. Preterm birth rate across maternal ethnicity in rural Yunnan, Southwest China, 2014-2018

Ethnicity	Rate per 100 deliveries (95% CI)				
	2014	2015	2016	2017	2018
Total	8.93(8.59, 9.27)	8.93(8.6, 9.26)	8.76(8.48, 9.05)	7.49(7.25, 7.72)	6.63(6.42, 6.83)
Han	8.22(7.81, 8.64)	7.8(7.41, 8.2)	8.16(7.8, 8.51)	6.93(6.64, 7.22)	6.1(5.84, 6.35)
Yi	8.05(7.3, 8.81)	6.86(6.17, 7.56)	7.01(6.35, 7.67)	6.62(6.01, 7.22)	5.87(5.32, 6.41)
Dai	11.96(10.1, 13.81)	15.19(13.2, 17.18)	12(10.46, 13.54)	10.26(8.97, 11.54)	7.5(6.48, 8.53)
Hani	11.73(9.4, 14.06)	11.87(9.64, 14.09)	8.47(6.73, 10.21)	6.42(5.06, 7.78)	4.99(3.99, 5.98)
Miao	11.36(8.94, 13.78)	10.21(8.22, 12.2)	14.3(12.04, 16.56)	13.43(11.64, 15.21)	14.83(13.12, 16.53)
Bai	12.76(9.64, 15.88)	14.95(11.4, 18.5)	10.83(8.62, 13.05)	6.34(4.98, 7.69)	3.71(2.78, 4.64)
Lisu	12.61(9.94, 15.27)	15.38(12.55, 18.22)	12.79(10.5, 15.09)	11.69(9.39, 13.98)	11.48(9.43, 13.53)
Zhuang	11.4(8.79, 14.01)	9.07(6.32, 11.82)	11.8(9.14, 14.45)	12.02(9.98, 14.07)	12.77(10.77, 14.76)
Hui	9.52(5.55, 13.49)	11.67(7.75, 15.6)	6.61(3.94, 9.27)	4.11(2.53, 5.69)	4.61(3.02, 6.19)
Wa	8.54(4.26, 12.81)	14.29(10.42, 18.15)	12.47(9.1, 15.84)	8.97(6.28, 11.65)	8.69(6.21, 11.17)
Lagu	9.02(3.93, 14.1)	16.33(11.15, 21.5)	15.38(11.46, 19.31)	13.39(10.42, 16.35)	9.29(7.02, 11.57)
Other	13.92(11.44, 16.4)	17.42(15.14, 19.7)	12.23(10.44, 14.02)	8.44(7.06, 9.82)	8.07(6.93, 9.2)
Total	8.93(8.59, 9.27)	8.93(8.6, 9.26)	8.76(8.48, 9.05)	7.49(7.25, 7.72)	6.63(6.42, 6.83)
Han	8.22(7.81, 8.64)	7.8(7.41, 8.2)	8.16(7.8, 8.51)	6.93(6.64, 7.22)	6.1(5.84, 6.35)
Yi	8.05(7.3, 8.81)	6.86(6.17, 7.56)	7.01(6.35, 7.67)	6.62(6.01, 7.22)	5.87(5.32, 6.41)

Note: CI: confidence interval.
